# Development of markers using microsatellite loci of two rove beetle species, *Paederus fuscipes* Curtis and *Aleochara* (*Aleochara*) *curtula* Goeze (Coleoptera: Staphylinidae), followed by analyses of genetic diversity and population structure

**DOI:** 10.1007/s13258-022-01293-2

**Published:** 2022-08-18

**Authors:** Yeon‐Jae Choi, Jeesoo Yi, Chan-Jun Lee, Ji-Wook Kim, Mi-Jeong Jeon, Jong-Seok Park, Sung-Jin Cho

**Affiliations:** 1 Department of Biological Sciences and Biotechnology, Chungbuk National University, Cheongju, 28644 Republic of Korea; 2 National Institute of Biological Resources, Environmental Research Complex, Incheon, 22689 Republic of Korea

**Keywords:** *Aleochara* (*Aleochara*) *curtula*, *Paederus fuscipes*, Microsatellite, Genetic diversity, Population structure, Korea

## Abstract

**Background:**

The family Staphylinidae is the most speciose beetle group in the world. The outbreaks of two staphylinid species, *Paederus fuscipes* and *Aleochara* (*Aleochara*) *curtula*, were recently reported in South Korea. None of research about molecular markers and genetic diversity have been conducted in these two species.

**Objective:**

To develop microsatellite markers and analyze the genetic diversity and population structures of two rove beetle species.

**Methods:**

NGS was used to sequence whole genomes of two species, *Paederus fuscipes* and *Aleochara* (*Aleochara*) *curtula*. Microsatellite loci were selected with flanking primer sequences. Specimens of *P*. *fuscipes* and *A*. *curtula* were collected from three localities, respectively. Genetic diversity and population structure were analyzed using the newly developed microsatellite markers.

**Results:**

The number of alleles ranged 5.727–6.636 (average 6.242) and 2.182–5.364 (average 4.091), expected heterozygosity ranged 0.560–0.582 (average 0.570) and 0.368–0.564 (average 0.498), observed heterozygosity ranged 0.458–0.497 (average 0.472) and 0.418–0.644 (average 0.537) in *P*. *fuscipes* and *A*. *curtula*, respectively. Population structure indicates that individuals of *A*. *curtula* are clustered to groups where they were collected, but those of *P*. *fuscipes* are not.

**Conclusion:**

Population structures of *P*. *fuscipes* were shallow. In *A*. *curtula*, however, it was apparent that the genetic compositions of the populations are different significantly depending on collection localities.

**Supplementary Information:**

The online version contains supplementary material available at 10.1007/s13258-022-01293-2.

## Introduction

Staphylinidae Latreille, the rove beetles, is one of the largest family represented by approximately 63,600 species (Irmler et al. 2018). Among 32 subfamilies of rove beetles, the subfamily Paederinae Fleming contains 5962 species within 225 genera (Anlaş and Çevik 2008). The genus *Paederus* Fabricius, a widespread speciose group, comprises approximately 530 described species, and 128 species in eight subgenera are recorded in Palaearctic region (Assing 2020). The subfamily Aleocharinae Fleming is the largest staphylinid subfamily including approximately 16,200 species (Leschen and Newton 2015). The genus *Aleochara* Gravenhorst is the one of the most speciose genera comprising about 540 species within 19 subgenera (Yamamoto and Maruyama 2016).

Recently, there have been nationwide or local outbreaks of *Paederus fuscipes* (available in: https://imnews.imbc.com/replay/2019/nwdesk/article/5522390_28802.html) and *Aleochara* (*Aleochara*) *curtula* (available in: https://www.kyongbuk.co.kr/news/articleView.html?idxno=2010335) in South Korea. These species are known as potential biocontrol agents because they feed on agricultural/hygienic pests. The adults of *Paederus* species are predators feeding on insect pests including moths, aphids and planthoppers (Khan et al. 2018; Nasir et al. 2012). Paederin, toxic compound synthesized by bacterial symbiont, is secreted from abdomen of *P*. *fuscipes* (Piel et al. 2004). Dermatitis symptoms by *P*. *fuscipes* and allied species have occurred worldwide (Gurcharan and Syed 2007; Kerion 1993; Todd et al. 1996). *Aleochara* females exclusively oviposit nearby pupated fly maggots, whose larvae trace and infiltrate into pupae (Maus et al. 1998). Although attacks to tourists caused by *A*. *curtula* have been reported in South Korea, damage in other countries is unknown.

The “Paederus dermatitis” damages have been reported continuously (Kim et al. 1989; Lim et al. 1996) and abundance of *A*. *curtula* also increased in 2018–2019. Despite their continuous outbreaks, studies of genetic diversity or genetic structure remain extremely limited. Microsatellites are DNA sequences consisting of 1–6 tandemly repeated nucleotides and can be used as informative DNA markers (Kwak et al. 2020). Several research about the genetic diversity of populations using microsatellite markers have been conducted for the use of conservation for endangered species (Kim et al. 2019; Kwak et al. 2020) or monitoring the domesticated organisms including crops and livestock (Biswas et al. 2020; Zhang et al. 2021).

In this study, we developed microsatellite markers of *P*. *fuscipes* and *A*. *curtula* for the first time. Genetic diversity was analyzed by genotyping polymorphic alleles using the newly developed microsatellite markers. We also visualized population structure. This work can provide insights for identifying characteristics of certain population within the species.

## Material and methods

### Collecting samples and DNA extraction

The specimens of *Paederus fuscipes* were collected manually on wet flatland including rice paddies from Taean-gun, Chungcheongnam-do [TA, 2.VII.2020], Suncheon-si, Jeollanam-do [SC, 21.V.2020], and Yesan-gun, Chungcheongnam-do [YS, 16.IV.2020]. Baited pitfall traps were used for sampling specimens of *Aleochara* (*Aleochara*) *curtula* from Muju-gun, Jeollabuk-do [MJ, 4–11.VI.2020], Gyeongju-si, Gyeongsangbuk-do [GJ, 20–27.VII.2018], and Jeongeup-si, Jeollabuk-do [JE, 1–8.IV.2020] (Table S1, Fig. 2). Genomic DNA samples were extracted from the entire body using the DNeasy^®^ Blood & Tissue Kit (Qiagen, Hilden, Germany).

### DNA sequencing and Microsatellite identification

After extracting the genomic DNA of *P. fuscipes* and *A. curtula*, we checked the quality of DNA by 1% agarose gel electrophoresis and PicoGreen^®^ dsDNA Assay (Invitrogen). DNA library was prepared according to Illumina Truseq DNA PCR-Free Library prep protocol. The quality of the libraries was verified by capillary electrophoresis (Bioanalyzer, Agilent). The library was clustered on the Illumina cBOT station and sequenced paired end for 101 cycles on the Novaseq 6000 sequencer according to the Illumina cluster and sequencing protocols.

Appropriate microsatellite loci from the genome sequence were searched using the QDD program (Meglécz et al. 2010), and flanking primer sets were also detected. Appropriate primer sequences were selected with high effective diversity according to the microsatellite development workflow (Lepais et al. 2020).

### Development of microsatellite markers

The forward primers used to amplify the microsatellite loci was labeled with a fluorescent dye, 6FAM. To validate the selected primers, Polymerase chain reaction (PCR) was performed in a total volume of 20 μl containing 0.4 μl genomic DNA, 8 μl each for forward and reverse primers and 10 μl BioFACT™ Lamp *Taq* PCR Master Mix 2 (BIOFACT, Daejeon, Korea), with an initial activation of 5 min at 95 °C, 30 cycles of 30 s at 95 °C, 30 s at the locus-specific annealing temperature, 30 s at 72 °C and a final extension at 72 °C for 5 min using a Thermal Cycler (VeritiPro, Marsiling, Singapore). Genotyping the obtained alleles was performed using ABI PRISM 3730XL Analyzer and GeneMapper^®^ Software Version 4.0.

### Data analysis

Genetic diversity parameters including the average number of alleles (Ad), expected heterozygosity (H_e_), observed heterozygosity (H_o_), and polymorphic information content (PIC) were analyzed using PowerMarker V3.25 (Liu and Muse 2005), which was also conducted for reconstructing UPGMA and NJ trees. GenAlEx 6.503 (Peakall and Smouse 2012) was used for AMOVA (analysis of molecular variance), PCoA (principal coordinates analysis), and calculating genetic distances (F_st_) among populations.

The population structure was analyzed using STRUCTURE 2.3.4 (Porras-Hurtado et al. 2013). To predict the optinum K value, the appropriate number of clusters constituting the structure, the program was run in three repetitions for each K from 2 to 10, with a burn-in period of 100,000 and 200,000 Markov chain Monte Carlo (MCMC) iterations. CONVERT (Glaubitz 2004) was used to transfer text files to input data for STRUCTURE 2.3.4. software. Appropriate K values were determined using the web-based software STRUCTURE HARVESTER (Earl and vonHoldt 2012).

## Results

### Development of the microsatellite markers

NGS produced 109,081,018 reads and 16,471,233,718 bp as total sequence from *Paederus fuscipes*. A total of 22,185,404,861 bp (146,923,211 reads) were obtained from *Aleochara* (*Aleochara*) *curtula*. The numbers of assembled contigs in *P*. *fuscipes* and *A*. *curtula* were 1,683,526 (totally 1,335,234,806 bp) and 63,105 (totally 128,993,284 bp), respectively. Eleven primers which amplified the gDNA of specimens were determined to be markers. The PIC (Polymorphism information content) value ranging 0.033–0.841 and 0.135–0.785 in *P*. *fuscipes* and *A*. *curtula*, respectively. The H_e_ (expected heterozygosity) ranged 0.033–0.856 (*P*. *fuscipes*) and 0.138–0.809 (*A*. *curtula*), H_o_ (observed heterozygosity) ranged 0.034–0.989 (*P*. *fuscipes*) and 0.122–0.989 (*A*. *curtula*). Developed markers were submitted to NCBI (Tables S2, S3).

### Genetic diversity

The Ad (the average number of alleles) of *P*. *fuscipes* based on developed 11 microsatellite markers ranged from 5.727 (TA) to 6.636 (YS), averaging 6.242. The Ad of *A*. *curtula* ranged from 2.182 (GJ) to 5.364 (MJ), averaging 4.091. The H_e_ of *P*. *fuscipes* and *A*. *curtula* ranged 0.560 (TA)—0.582 (SC) and 0.368 (GJ)—0.564 (MJ), respectively The H_o_ of *P*. *fuscipes* and *A*. *curtula* ranged 0.458 (SC)—0.497 (TA) and 0.418 (GJ)—0.644 (JE), respectively. (Table 1). UPGMA phylogenetic analysis suggest that each population has not been clustered as clades in *P*. *fuscipes* (Figure S1a). Populations of *A*. *curtula* have been clustered depending on localities collected (Figure S1c). Neighbor-joining analysis also resulted correspond to the UPGMA (Figure S1b, d).

**Table 1:** Genetic diversity of Paederus fuscipes and Aleochara (Aleochara) curtula populations

Species	ID	N	Ad	H_e_	H_o_
*P*. *fuscipes*	TA	30	5.727	0.560	0.497
	SC	30	6.364	0.582	0.458
	YS	29	6.636	0.569	0.460
	Average		6.242	0.570	0.472
*A*. *curtula*	MJ	30	5.364	0.564	0.551
	JE	30	4.727	0.563	0.644
	GJ	30	2.182	0.368	0.418
	Average		4.091	0.498	0.537

*N* number of samples, *Ad* mean number of alleles, *H*_*e*_ expected heterozygosity, *H*_*o*_ observed heterozygosity

### Population structure

**Fig. 1:**
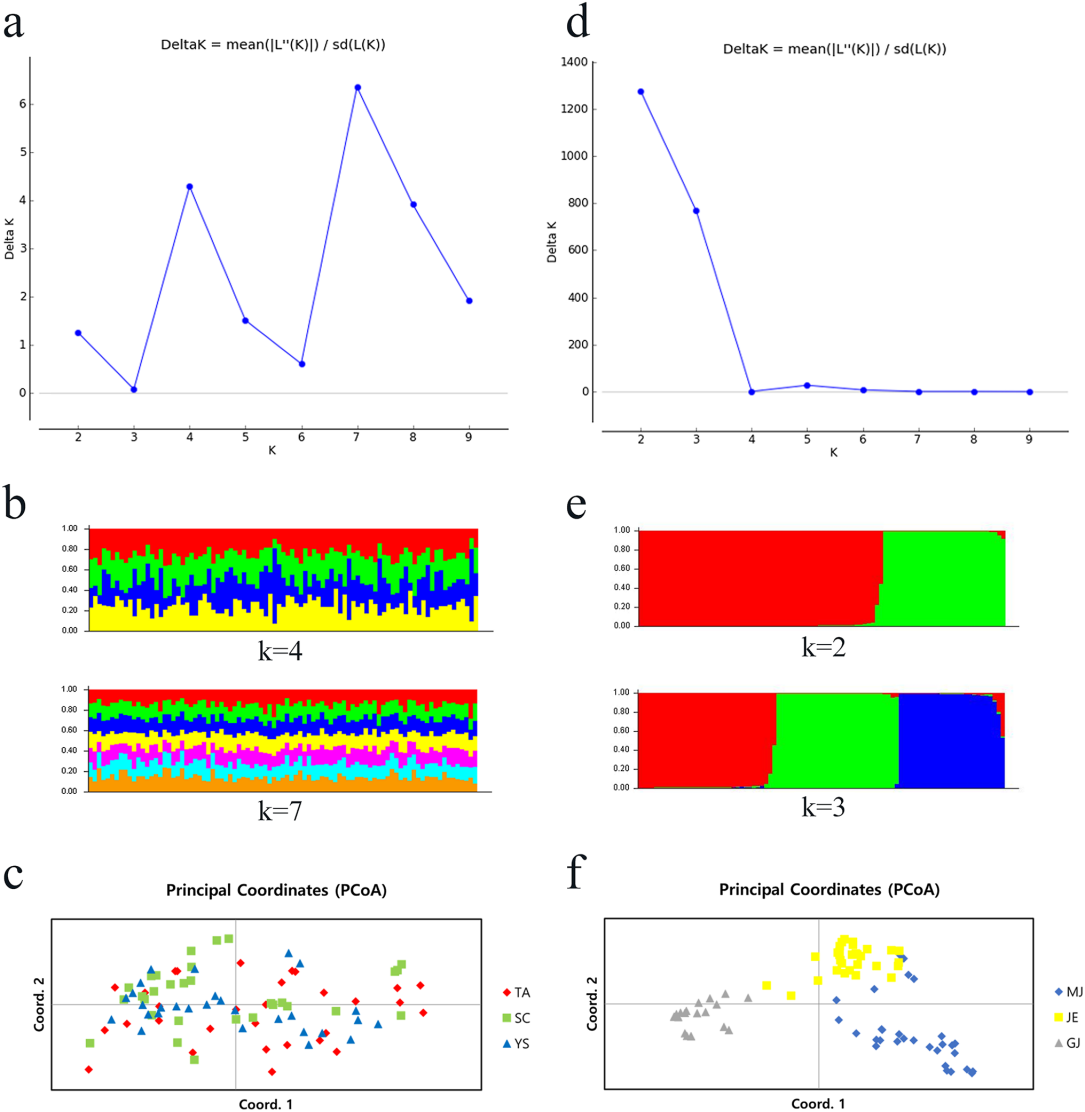
Population structures. **a**, **d** Appropriate K values calculated by STRUCTURE HARVESTER. **b**, **e** Estimated population structures by STRUCTURE. **c**, **f** PCoA results. **a**–**c** *Paederus fuscipes*. **d**–**f** *Aleochara* (*Aleochara*) *curtula*

**Fig. 2:**
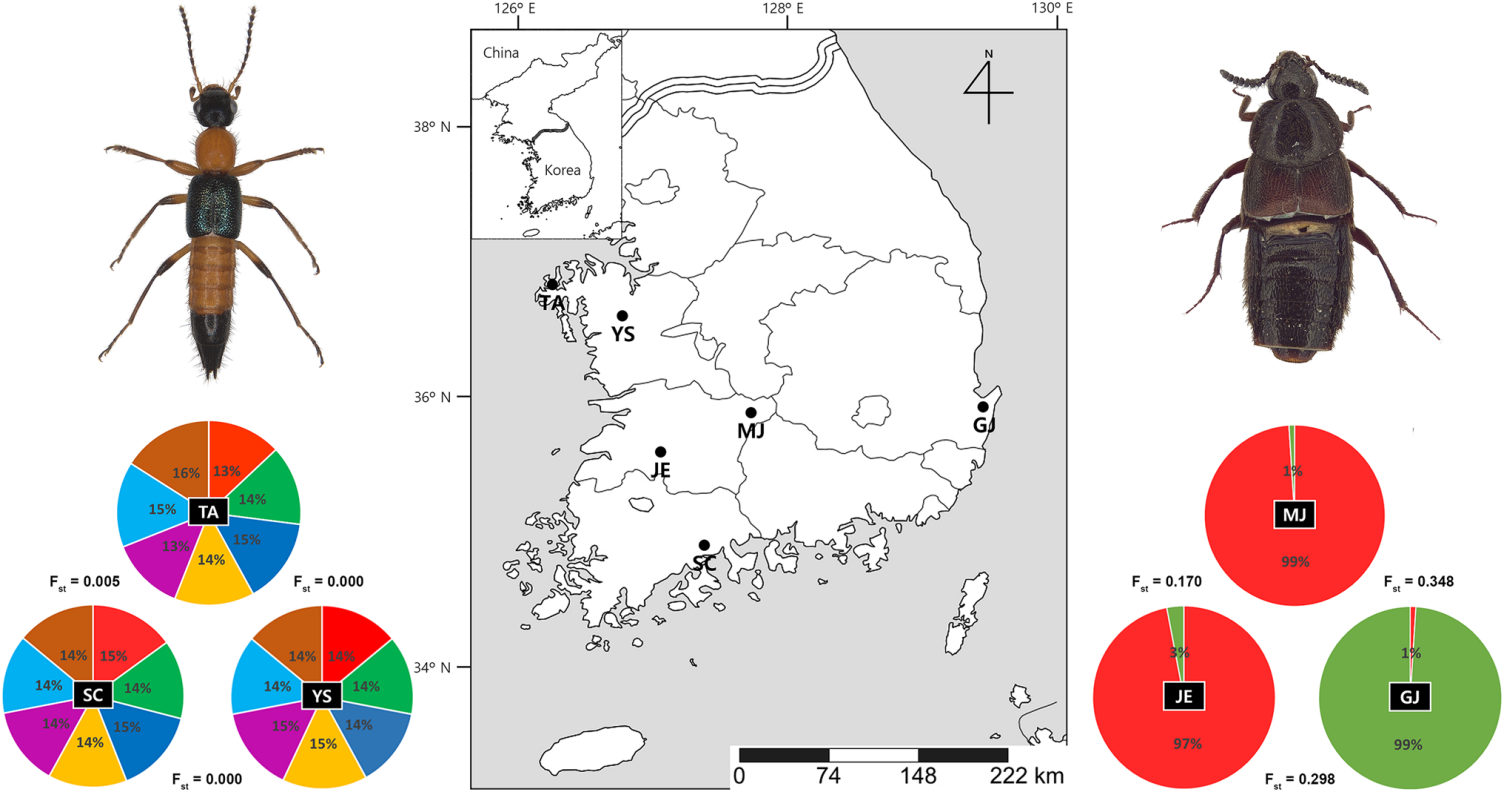
The average cluster assignments at each location and genetic distances among populations. Habitus of examined species are provided. Left: *Paederus fuscipes*. Right: *Aleochara* (*Aleochara*) *curtula*. Genetic structures are displayed as pie charts. F_st_ values between TA–YS and SC–YS was calculated to be 0 because of a few individual samples

Based on STRUCTURE HARVERSTER, appropriate clusters were determined to be K = 7 or K = 4 in *P*. *fuscipes* and K = 2 or K = 3 in *A*. *curtula*, respectively (Fig. 1a, d). The structure was not divided separately neither K = 7 nor K = 4 in *P*. *fuscipes* (Fig. 1b). However, that was divided into two (K = 2) or three (K = 3) groups in *A*. *curtula* (Fig. 1e). PCoA also shows scattered plots for *P*. *fuscipes* but clustered plots for *A*. *curtula* (Fig. 1c, f). The results of AMOVA suggests relatively high genetic differentiation with 26% of variation among populations of *A*. *curtula*, while *P*. *fuscipes* shows low population differentiation (Table 2). The highest genetic distance (F_st_) was 0.005 between TA and SC in *P*. *fuscipes*, 0.348 between MJ and GJ in *A*. *curtula* (Fig. 2).

**Table 2:** AMOVA result of two species

Species	Source	df	SS	MS	Est. Var	%
*P*. *fuscipes*	Among Pops	2	8.230	4.115	0.005	0%
	Among Indiv	86	326.284	3.794	0.593	18%
	Within Indiv	89	232.156	2.608	2.608	81%
	Total	177	566.671		3.207	100%
*A*. *curtula*	Among Pops	2	118.411	59.206	0.946	26%
	Among Indiv	87	210.4	2.418	0	0%
	Within Indiv	90	241	2.678	2.678	74%
	Total	179	569.811		3.624	100%

*df* degree of freedom, *SS* sum of squares, *MS* mean squares

## Discussion

We amplified 11 microsatellite loci from *Paederus fuscipes* and *Aleochara* (*Aleochara*) *curtula*, respectively. Among the newly developed markers, PF-005, PF-006, AC-002 are weakly informative (PIC < 0.25), PF-001 is quite informative (0.25 < PIC < 0.5), and the others are highly informative (PIC > 0.5) (Tables S2, S3) according to Botstein et al. (1980).

The H_e_ of SC was relatively higher than the other populations, and GJ was the minimum. The minimum H_o_ was GJ, however, the maximum was JE (Table 1). Considering the H_e_ is calculated theoretically assuming random mating, it cannot be able to exactly match or corresponding the heterozygosity within actual populations and gene diversity (Gregorius 1978).

Genetic diversity among populations of *P*. *fuscipes* was almost identical and it showed shallow structure both K = 7 and K = 4 (Fig. 1b). Although the physical distances among three localities, genetic distances were extremely low (Fig. 2). It suggests that gene flows of *P*. *fuscipes* individuals were less limited and there were robust genetic communications. Populations of *A*. *curtula* showed strong structure. Estimated K value supported two genetic groups (Fig. 1d, e), which coincide with the pie chart of Fig. 2 (right). Especially GJ (Gyeongju), the region where the outbreak occurred, indicated low genetic diversity and extremely high genetic distances against the others.

## Conclusion

Geographically isolated, individuals of GJ are restricted to communicate with those of other localities. In addition, the evolution within genus *Aleochara* took place rapidly. Molecular phylogeny suggested that the diverse species originated in relatively short period (Maus et al. 2001). Our results provided basic data for a quick monitoring technique dealing with future outbreaks, but also discovering potential subspecies of *A*. *curtula*. It is expected that microsatellite markers can be applied to evaluate genetic diversity of more than population level within the family Staphylinidae.

## Supplementary Information

Below is the link to the electronic supplementary material.

Supplement figure 1. Phylogenetic tree based on UPGMA and Neighbor-joining analyses (TIF 3535 KB)

Supplementary file2 (DOCX 16 KB)

Supplementary file3 (DOCX 23 KB)

Supplementary file4 (DOCX 23 KB)
