# Supplementary figures and images for: Development of markers using microsatellite loci of two rove beetle species, Paederus fuscipes Curtis and Aleochara (Aleochara) curtula Goeze (Coleoptera: Staphylinidae), followed by analyses of genetic diversity and population structure
Source: Genes Genomics. 2022 Aug 18;44(12):1471–6. doi: 10.1007/s13258-022-01293-2 (PMC9684238; doi:10.1007/s13258-022-01293-2)

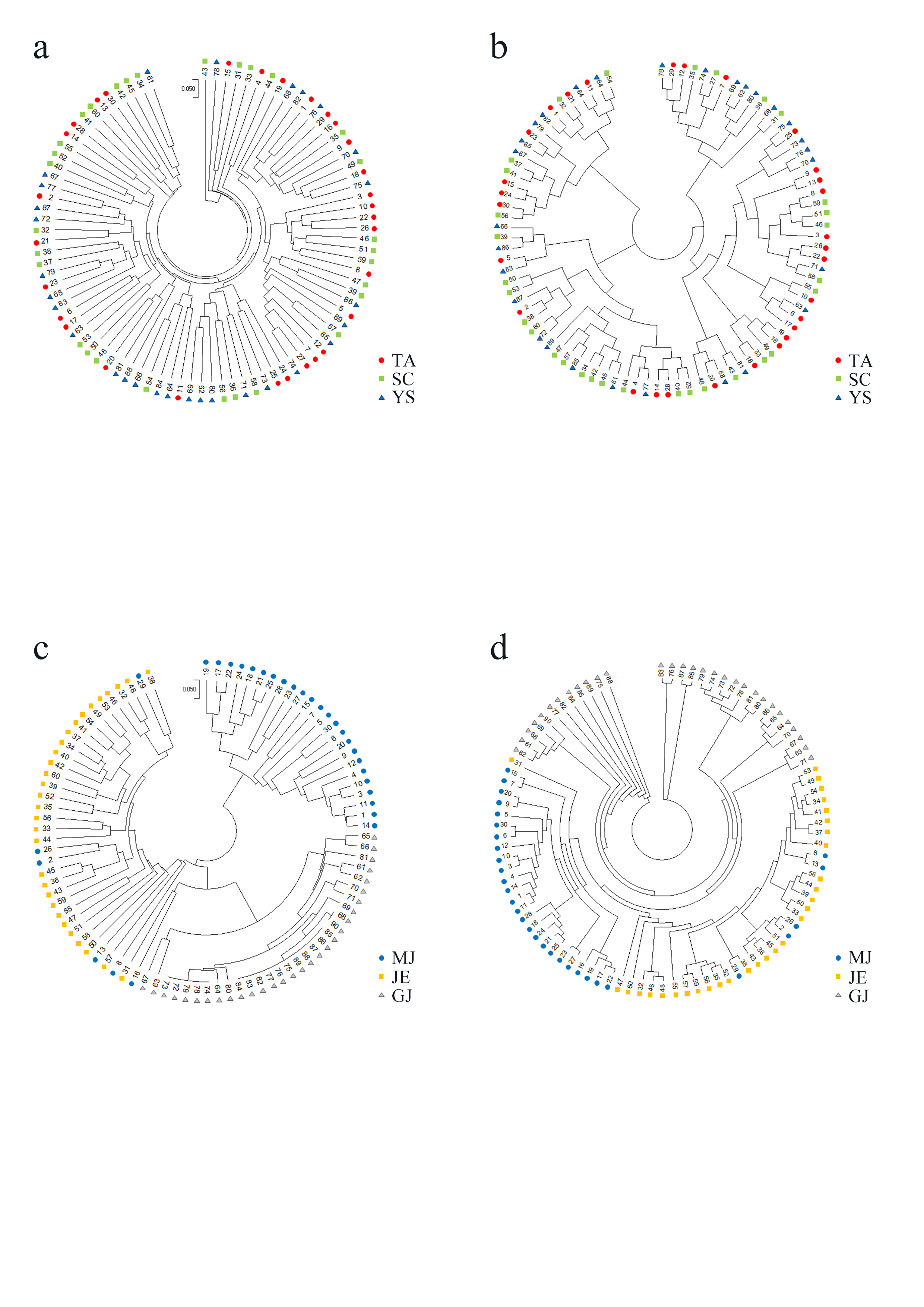

Supplement: Supplementary file 1 — Supplement figure 1. Phylogenetic tree based on UPGMA and Neighbor-joining analyses (TIF 3535 KB) [file 13258_2022_1293_MOESM1_ESM.tif]
