# Supplementary material for: Development of markers using microsatellite loci of two rove beetle species, Paederus fuscipes Curtis and Aleochara (Aleochara) curtula Goeze (Coleoptera: Staphylinidae), followed by analyses of genetic diversity and population structure
Source: Genes Genomics. 2022 Aug 18;44(12):1471–6. doi: 10.1007/s13258-022-01293-2 (PMC9684238; doi:10.1007/s13258-022-01293-2)
Supplement: Supplementary file 2 — Supplementary file2 (DOCX 16 KB) [file 13258_2022_1293_MOESM2_ESM.docx]

| Species | ID | Sample locality | Lat. (ºN)/long. (ºE)/alt. (meter) | No. individuals |
| --- | --- | --- | --- | --- |
| *P*. *fuscipes* | TA | Taean-gun, Chungcheongnam-do, Republic of Korea | 36°50'41.5"/126°11'45.8"/7 | 30 |
|  | SC | Suncheon-si, Jeollanam-do, Republic of Korea | 34°53'36.16"/127°30'37.15"/1 | 30 |
|  | YS | Yesan-gun, Chungcheongnam-do, Republic of Korea | 36°33'50.4"/126°48'16.32"/41 | 29 |
| *A*. *curtula* | MJ | Muju-gun, Jeollabuk-do, Republic of Korea | 35°56'52.6"/127°41'40.4"/884 | 30 |
|  | JE | Jeongeup-si, Jeollabuk-do, Republic of Korea | 35°35'02.22"/127°03'59.91"/171 | 30 |
|  | GJ | Gyeongju-si, Gyeongsangbuk-do, Republic of Korea | 35°47'20.20"/129°20'10.40"/282 | 30 |

Table S1. Collection localities.
