# Supplementary material for: Development of markers using microsatellite loci of two rove beetle species, Paederus fuscipes Curtis and Aleochara (Aleochara) curtula Goeze (Coleoptera: Staphylinidae), followed by analyses of genetic diversity and population structure
Source: Genes Genomics. 2022 Aug 18;44(12):1471–6. doi: 10.1007/s13258-022-01293-2 (PMC9684238; doi:10.1007/s13258-022-01293-2)
Supplement: Supplementary file 3 — Supplementary file3 (DOCX 23 KB) [file 13258_2022_1293_MOESM3_ESM.docx]

| Locus | GenBank No. | Primer sequence (5'-3') | Repeat motif | Dye | T_a_ (°C) | K | Size range | H_e_ | H_o_ | PIC |
| --- | --- | --- | --- | --- | --- | --- | --- | --- | --- | --- |
| PF-001 | MW373088 | F: GCGCGAAGGATTAACTCAGG | (AAAT)_10_ | 6FAM | 58.5 | 3 | 109-121 | 0.516 | 0.989 | 0.399 |
|  |  | R: ATAAGTTCTGGCGGGCTTCC |  |  |  |  |  |  |  |  |
| PF-002 | MW373089 | F: CGGCTTCGAGTTTGCTTTCC | (AC)_9_ | 6FAM | 58.5 | 10 | 124-144 | 0.706 | 0.517 | 0.669 |
|  |  | R: CCGACATCGAGGAAGTTGCT |  |  |  |  |  |  |  |  |
| PF-003 | MW373090 | F: ATACAGACGCCGCATTTCCC | (AC)_8_ | 6FAM | 58.5 | 12 | 181-203 | 0.856 | 0.36 | 0.841 |
|  |  | R: TTAGCAGGGTGGGTGCATAC |  |  |  |  |  |  |  |  |
| PF-004 | MW373091 | F: CCACTTAACCCTCGGACGTG | (AC)_8_ | 6FAM | 59.5 | 14 | 118-148 | 0.635 | 0.539 | 0.614 |
|  |  | R: CCGTGTTCCTCCCGCATTTA |  |  |  |  |  |  |  |  |
| PF-005 | MW373092 | F: ACAGGCACAACGATTGCTAG | (AACCT)_8_ | 6FAM | 57.5 | 5 | 78-88 | 0.033 | 0.034 | 0.033 |
|  |  | R: TATTGCAGCTGACCTGGCTC |  |  |  |  |  |  |  |  |
| PF-006 | MW373093 | F: TCTCGAATTTCCAGGGCACG | (AG)_7_ | 6FAM | 57.5 | 4 | 176-184 | 0.224 | 0.157 | 0.212 |
|  |  | R: TCACAACTCTGTCTGCAGCA |  |  |  |  |  |  |  |  |
| PF-007 | MW373094 | F: ACCGCTTTCTACAGCATCCC | (AAAT)_7_ | 6FAM | 58.5 | 9 | 162-174 | 0.595 | 0.618 | 0.535 |
|  |  | R: AGAGCAGGGACATGTGGTTG |  |  |  |  |  |  |  |  |
| PF-008 | MW373095 | F: TACGTAGTGTTCGACCCTCG | (AG)_7_ | 6FAM | 58.5 | 12 | 164-178 | 0.774 | 0.588 | 0.748 |
|  |  | R: ATTACGGGACCACGTTTCGG |  |  |  |  |  |  |  |  |
| PF-009 | MW373096 | F: TGCCTTCGATTGAAACGTCG | (AC)_7_ | 6FAM | 56.5 | 6 | 171-203 | 0.595 | 0.337 | 0.52 |
|  |  | R: TGCTGCGGATTTGCCAGATA |  |  |  |  |  |  |  |  |
| PF-010 | MW373097 | F: CCAAGGCAATTGTTCAGCGT | (AG)_7_ | 6FAM | 56.5 | 11 | 109-119 | 0.801 | 0.605 | 0.78 |
|  |  | R: AAGGTCGCTCGTTAACTGGA |  |  |  |  |  |  |  |  |
| PF-011 | MW373098 | F: TCCTGATTATGCCTGCCAGA | (AC)_7_ | 6FAM | 56.5 | 8 | 120-140 | 0.633 | 0.516 | 0.59 |
|  |  | R: TTCGATCGCCCTTCTGCAAT |  |  |  |  |  |  |  |  |

Table S2. Characteristics of the 11 polymorphic microsatellite markers from *Paederus fuscipes*

T_a_: Annealing temperature, K: Number of alleles, H_o_: Observed heterozygosity, H_e_: Expected heterozygosity, PIC: Polymorphic Information Content
