# Supplementary material for: Development of markers using microsatellite loci of two rove beetle species, Paederus fuscipes Curtis and Aleochara (Aleochara) curtula Goeze (Coleoptera: Staphylinidae), followed by analyses of genetic diversity and population structure
Source: Genes Genomics. 2022 Aug 18;44(12):1471–6. doi: 10.1007/s13258-022-01293-2 (PMC9684238; doi:10.1007/s13258-022-01293-2)
Supplement: Supplementary file 4 — Supplementary file4 (DOCX 23 KB) [file 13258_2022_1293_MOESM4_ESM.docx]

| Locus | GenBank No. | Primer sequence (5'-3') | Repeat motif | Dye | T_a_ (°C) | K | Size range | H_e_ | H_o_ | PIC |
| --- | --- | --- | --- | --- | --- | --- | --- | --- | --- | --- |
| AC-001 | MW373077 | F: GCACGTTTGTTCGTTGAGCA | (AGC)_12_ | 6FAM | 57.5 | 7 | 118-169 | 0.753 | 0.345 | 0.713 |
|  |  | R: CGGCGTGAGGAAGTCAAACT |  |  |  |  |  |  |  |  |
| AC-002 | MW373078 | F: CAAAGCCGCTAGAGCTCTGT | (AC)_10_ | 6FAM | 58.5 | 5 | 100-146 | 0.138 | 0.122 | 0.135 |
|  |  | R: GCTCTCGTTTCCCTTTCTGC |  |  |  |  |  |  |  |  |
| AC-003 | MW373079 | F: GGTGAACTCGACGTACGCAT | (AC)_9_ | 6FAM | 58.5 | 7 | 101-199 | 0.602 | 0.989 | 0.522 |
|  |  | R: CAGCCGGCACTGCAATTATC |  |  |  |  |  |  |  |  |
| AC-004 | MW373080 | F: GTCGACAAACACGATGCGTC | (AGGC)_9_ | 6FAM | 58.5 | 9 | 102-162 | 0.62 | 0.562 | 0.588 |
|  |  | R: GCGAAGGGTGAAATGAAGCG |  |  |  |  |  |  |  |  |
| AC-005 | MW373081 | F: GTCCTGCCTTCCTGTCACTG | (CG)_9_ | 6FAM | 59.5 | 7 | 102-178 | 0.791 | 0.652 | 0.758 |
|  |  | R: CCAATGCCCTCCAATTCCCA |  |  |  |  |  |  |  |  |
| AC-006 | MW373082 | F: GGGAATTGCGTGCGATGTTT | (CG)_8_ | 6FAM | 57.5 | 13 | 101-171 | 0.809 | 0.556 | 0.785 |
|  |  | R: GGCTGACAGGTATTTGGCGA |  |  |  |  |  |  |  |  |
| AC-007 | MW373083 | F: ACATCGAGGAAACTGCGTGA | (AGC)_8_ | 6FAM | 56.5 | 5 | 107-179 | 0.688 | 0 | 0.653 |
|  |  | R: GGTTTGCGCTGCTGTGTATT |  |  |  |  |  |  |  |  |
| AC-008 | MW373084 | F: ATGGCTCACCGTACTCGAAC | (AAC)_8_ | 6FAM | 57.5 | 8 | 103-187 | 0.796 | 0.929 | 0.768 |
|  |  | R: GCACTTGATTCACGACTGCA |  |  |  |  |  |  |  |  |
| AC-009 | MW373085 | F: CCCATGCTCTAGCTGTTGGT | (AAC)_7_ | 6FAM | 58.5 | 7 | 101-137 | 0.725 | 0.787 | 0.688 |
|  |  | R: TGCGGACGAAGAGTGGTTAG |  |  |  |  |  |  |  |  |
| AC-010 | MW373086 | F: ATGGGCGTGTTGTTCACTCA | (AAG)_7_ | 6FAM | 57.5 | 6 | 127-202 | 0.759 | 0.556 | 0.727 |
|  |  | R: GACCGCTTCTTGTATCGCCT |  |  |  |  |  |  |  |  |
| AC-011 | MW373087 | F: CCGTCCGAATCCAGAACCAA | (AG)_7_ | 6FAM | 59.5 | 10 | 109-193 | 0.771 | 0.775 | 0.74 |
|  |  | R: GTGAGGTGCCATACAGGGAG |  |  |  |  |  |  |  |  |

Table S3. Characteristics of the 11 polymorphic microsatellite markers from *Aleochara curtula*

T_a_: Annealing temperature, K: Number of alleles, H_o_: Observed heterozygosity, H_e_: Expected heterozygosity, PIC: Polymorphic Information Content
